# One-Dimensional Organic–Inorganic Material (C_6_H_9_N_2_)_2_BiCl_5_: From Synthesis to Structural, Spectroscopic, and Electronic Characterizations

**DOI:** 10.3390/ijms22042030

**Published:** 2021-02-18

**Authors:** Hela Ferjani, Hammouda Chebbi, Mohammed Fettouhi

**Affiliations:** 1 Chemistry Department, College of Science, IMSIU (Imam Mohammad Ibn Saud Islamic University), Riyadh 11623, Saudi Arabia; 2 Laboratory of Materials, Crystal Chemistry and Applied Thermodynamics, Faculty of Sciences of Tunis, University of Tunis El Manar, 2092 El Manar II, Tunisia; chebhamouda@yahoo.fr; 3 Department of Chemistry, King Fahd University of Petroleum and Minerals, Dhahran 31261, Saudi Arabia; fettouhi@kfupm.edu.sa

**Keywords:** crystal structure, chlorobismuthate, vibrational properties, Hirshfeld surface analysis, DFT, optical properties, electronic characterization

## Abstract

The new organic–inorganic compound (C_6_H_9_N_2_)_2_BiCl_5_ (I) has been grown by the solvent evaporation method. The one-dimensional (1D) structure of the allylimidazolium chlorobismuthate (I) has been determined by single crystal X-ray diffraction. It crystallizes in the centrosymmetric space group C2/c and consists of 1-allylimidazolium cations and (1D) chains of the anion BiCl_5_^2−^, built up of corner-sharing [BiCl_6_^3−^] octahedra which are interconnected by means of hydrogen bonding contacts N/C–H⋯Cl. The intermolecular interactions were quantified using Hirshfeld surface analysis and the enrichment ratio established that the most important role in the stability of the crystal structure was provided by hydrogen bonding and H···H interactions. The highest value of E was calculated for the contact N⋯C (6.87) followed by C⋯C (2.85) and Bi⋯Cl (2.43). These contacts were favored and made the main contribution to the crystal packing. The vibrational modes were identified and assigned by infrared and Raman spectroscopy. The optical band gap (Eg = 3.26 eV) was calculated from the diffuse reflectance spectrum and showed that we can consider the material as a semiconductor. The density functional theory (DFT) has been used to determine the calculated gap, which was about 3.73 eV, and to explain the electronic structure of the title compound, its optical properties, and the stability of the organic part by the calculation of HOMO and LUMO energy and the Fukui indices.

## 1. Introduction

Halometallates (III) organic-inorganic materials have generated significant interest because of their special structural features and high potential applications, such as electronic and optical materials [1,2,3,4,5,6]. Among these classes of materials, halogeno bismuthate(III) with general formula A_a_B_b_X_3b+a_, where A = organic amine and X = I, Br, Cl, have emerged as an up-and-coming class of luminescent and semiconducting materials and have stimulated considerable interest among researchers [7,8,9,10,11,12]. The anion substructure of these materials comprises MX_6_ octahedra that may be isolated (zero-dimensional—0D) or linked by edges, corners or faces (1D, 2D, 3D architectures) [13,14,15]. Low dimensional (1D and 0D) substructures expand physical properties associated with organic and inorganic constituents. The anionic parts act as semiconductors and the organic cations act as potential barriers, contributing to original electronic and optical behavior [16,17,18]. Thus, in order to increase the dimensionality of the inorganic substructures and decrease the band gap, an additional successful scheme is the incorporation of multifunctional organic cations capable of influencing the bonding structures within the inorganic network and increasing the orbital interaction across adjacent chains. The organic cations control the structure through their size, in addition to hydrogen bonds and/or van der Waals interactions. Generally, the trend is that non-bulky cations with a delocalized charge, such as imidazolium groups, can form hydrogen bonds with the terminal nitrogen atoms (N) and lead to anions with high N/M ratios (M: post-transition metals), as in the case of 1D M^III^X_5_^2−^.

In the present work, we describe the synthesis and experimental characterization supported with theoretical studies of a material of formula (C_6_H_9_N_2_)_2_BiCl_5_. The results of the vibrational (Raman–infrared), optical (absorption-band gap and HOMO–LUMO transitions) properties, and Hirshfeld surface analysis are discussed. The density functional theory DFT calculation was used to explain the optical properties by determining the calculated gap, the dielectric function, and the refractive index. The title compound stability is discussed in terms of the total and partial density of states and the HOMO/LUMO quantum mechanical descriptors of the organic part. The calculated Fukui indices give an insight into the nucleophilic and electrophilic sites of the organic component.

## 2. Experimental and Computational Methods

### 2.1. Synthesis of (C_6_H_9_N_2_)_2_BiCl_5_

Bismuth(III) oxide (Bi_2_O_3_), hydrochloric acid (HCl; 37% in water), and 1-Allylimidazole (C_6_H_8_N_2_) were purchased from Sigma-Aldrich and used without further purification. The synthesis of (C_6_H_9_N_2_)_2_BiCl_5_ material was carried out by slow evaporation method at room temperature. Values of 1.0 mmol (0.65 g) of Bi_2_O_3_ and 2.0 mmol (0.30 g) of 1-Allylimidazole were dissolved together in 20 mL of an aqueous hydrochloric acid solution (pH ≈ 3). The mixture was stirred with heating (50 °C) for one hour. Then, it was covered with paraffin film and allowed to evaporate at room temperature. Colorless block crystals with suitable dimensions for crystallographic study were formed after 10 days.

### 2.2. Surface Investigation by SEM/EDX

SEM-EDX was used to investigate the morphology and the elemental composition of the crystals of the title compound. SEM images were acquired using a JEOL, JSM-6380-LA SEM-EDXS spectrometer, typically operated with an acceleration voltage of 15 kV coupled with an energy-dispersive X-ray spectrometry (EDX) detector system. In order to limit detrimental charge effects, a platinum-rich tape was used to partially cover the sample.

### 2.3. X-Ray Diffraction Analysis

Single crystal X-ray data were collected on a Bruker D8 Quest diffractometer (MoKα radiation λ = 0.71073 Å) at 298 K using Bruker APEX3 software package [19]. Data reduction was performed using SAINT [20]. Multi-scan absorption correction was performed using SADABS [21]. The structures were solved by direct methods with SHELXS using SHELXTL package and refined using full-matrix least-squares procedures on F2 via the program SHELXL-2014 [22]. All non-hydrogen atoms were refined with anisotropic displacement parameters. Hydrogen atoms were placed at calculated positions using a riding model. Molecular graphics were prepared using Diamond 3 [23]. Details of the data collection and crystallographic parameters are given in Table 1. Selected interatomic distances and bond angles are presented in Table 2. Additional information on the crystal structure study can be obtained in the form of a crystallographic information file (CIF), which was deposited in the Cambridge Crystallographic Data Center (CCDC) database (deposition number 1903636).

For the other physical characterizations of the title compound, the single crystals have been selected according to their morphologies and color under binocular glass. Then, the single crystals were ground by means of an agate mortar to obtain a polycrystalline powder. The powder’s purity has been verified by the powder X-ray diffraction (PXRD) followed by Rietveld refinement.

**Figure 1:**
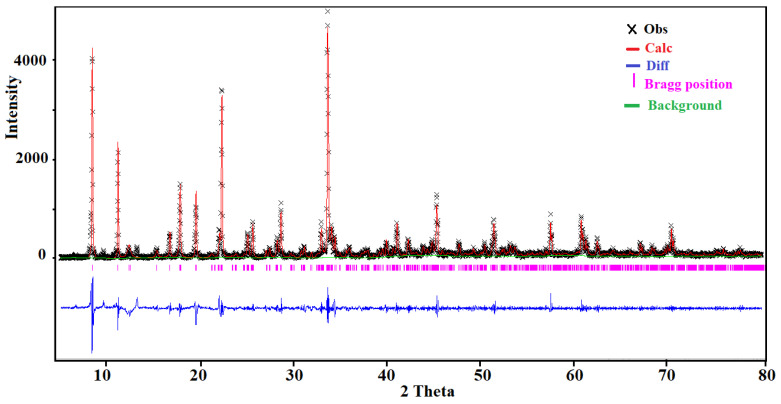
The Rietveld refinement of the room temperature powder diffraction pattern of (I).

The PXRD pattern was recorded, at room temperature, using a Bruker D8 ADVANCE diffractometer equipped with a Cu anode (CuKα radiation λ = 1.54056 Å). The measurements were performed in the range 5–80° under Bragg–Brentano geometry with a step of 0.02° and a counting time of 2 s per step. The Rietveld refinement was performed by using the GSAS computer program [24,25]. The crystallographic data of the single crystal have been used as a starting set. The results of the Rietveld refinement are depicted in Figure 1. The final agreement factors were R_p_ = 0.042, R_wp_ = 0.126, and χ^2^ = 0.051. The structural model was fully consistent with the one obtained from the single crystal X-ray diffraction data.

### 2.4. Physical Measurements

The solid-state infrared spectrum of (C_6_H_9_N_2_)_2_BiCl_5_ was obtained using a Nicolet NXR FTIR spectrometer, at room temperature, on KBr pellets ranging from 400 to 4000 cm^−1^. We recorded the Raman spectrum using a HORIBA Lab RAM HR Evolution Surface-Enhanced Raman Scattering Microscope in the 400–50 cm^−1^ region. Room-temperature diffuse absorption and diffuse reflectance spectra of polycrystalline powder of (C_6_H_9_N_2_)_2_BiCl_5_ were measured using a JASCO V-770 spectrophotometer in the wavelength range 300–700 nm, and BaSO_4_ plates were used as a reference.

### 2.5. Computational Methods

The structural model obtained from the X-ray single-crystal refinement has been used as a starting model in the density functional theory (DFT) calculation. The electronic structure and the optical property parameters were obtained using the electron exchange-correlation functional proposed by Perdew–Burke–Ernzerhof (PBE) [26] and the projector augmented-wave (PAW) pseudopotential plane-wave method, as implemented in the CASTEP code [27]. A plane-wave basis set was adopted with an energy cutoff of 600 eV. The atomic force, maximum displacement, and total energy convergence criteria were 0.05 eV Å^−1^, 0.002 Å, and 10^−6^ eV, respectively. The limited memory BFGS method has been used for energy minimization. We carried out the Brillouin-zone integrations using the Monkhorst–Pack scheme [28], with a regularly spaced mesh of 2 × 2 × 2 points in the reciprocal unit cell.

The HOMO, LUMO, and Fukui indices of the organic part of the title compound have been obtained using DMol3 code [29]. The calculations were carried out through Mulliken population analysis [30] and using the PBE functional method with DNP basis set [31]. The convergence parameters were as follows: maximum displacement of 0.005 Å, SCF tolerance 1 × 10^−6^ eV/atom, convergence energy tolerance 1 × 10^−6^ Ha. After the geometry optimization convergence, the following parameters were calculated: the HOMO (highest occupied molecular orbital energy), LUMO (lowest unoccupied molecular orbital energy) [32], and Fukui indices (FI) [33].

We drew the Hirshfeld Surfaces [34] and their relative 2D fingerprint plots [35] using Crystal Explorer 17 software [36] with a final refined crystallographic information file as the input. The quantifying and decoding of the intercontact in the molecular packing were created using d_norm_ (normalized contact distance) and 2D fingerprint plots, respectively. The dark-red spots on the d_norm_ surface resulted from the short interatomic contacts, while the other intermolecular interactions appeared as light-red spots. The d_i_ (inside) and d_e_ (outside) represents the distances to the Hirshfeld surface from the nuclei, with respect to the relative van der Waals radii. The proportional contribution of intercontact over the surface was visualized by the color gradient (blue to red) in the fingerprint plot. The enrichment ratios Exy were obtained from the actual contacts between the different chemical species (x, y) and equiprobable proportions calculated from the surface chemical content [37,38]. An enrichment ratio larger than unity indicated that the contact type was favored and made the largest contribution to the crystal packing.

## 3. Discussion

### 3.1. Energy-Dispersive X-ray Analysis (EDX)

**Figure 2:**
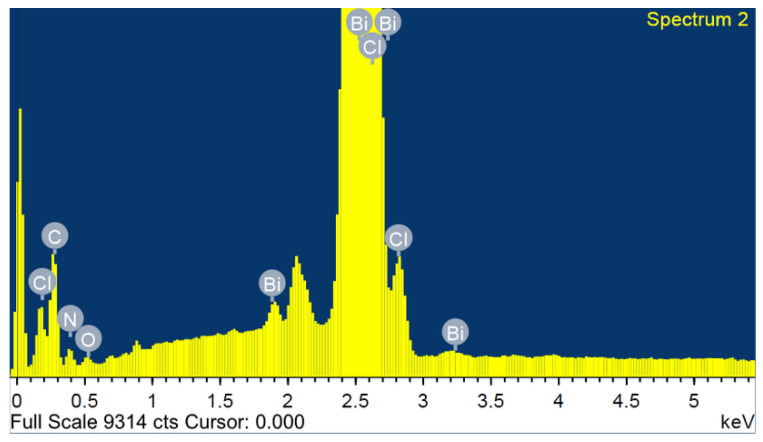
EDX analysis of (I).

The SEM image and the characteristic EDX spectrum are shown in Figure 2. The SEM micrograph (Figure S1) shows a good crystal quality, with a width of 4.5 mm. Figure 2 shows the total area-averaged EDX result obtained for the synthesized (C_6_H_9_N_2_)_2_BiCl_5_. Characteristic X-ray peaks associated with C, Bi, O, N and Cl are all identified and labeled in Figure 2. Each element’s weight percentage was determined to be 32.51% carbon, 32.98% bismuth, 1.37% oxygen, 8.42% nitrogen, and 24.72% chlorine. The small weight percent, accounted for by the small oxygen peak visible in the EDX spectrum, is attributed to the impurity acquired during the synthesis or the coating processes.

### 3.2. Crystal Structure Description

**Figure 3:**
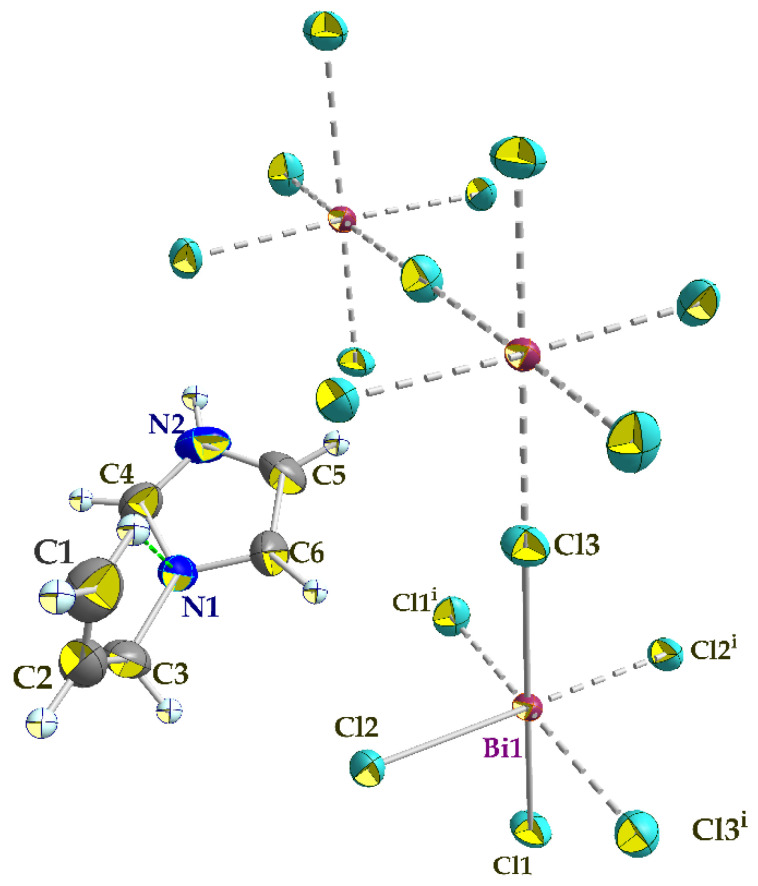
An ORTEP plot of the molecular entities of (I), showing the atom numbering scheme. Anisotropic displacement parameters are shown at the 50% probability level. The grey dashed lines present bonds between atoms generated by symmetry elements (i) −*x* + 1, *y*, −*z* + 1/2. The intra-molecular hydrogen bond within the cation is shown in a green dashed line.

The single crystal X-ray diffraction analysis showed that the title compound (C_6_H_9_N_2_)_2_BiCl_5_, (I), crystallizes in the centrosymmetric space group C2/c and the asymmetric unit comprises one Bi^3+^ cation (located on a crystallographic two-fold axis), three chloride anions (one of which is located in a crystallographic inversion center), and one 1-allylimidazolium cation (Figure 3). The cations are related by glide planes and an inversion center. The Bi^3+^ ion is coordinated by six chloride anions in a distorted octahedral geometry.

**Figure 4:**
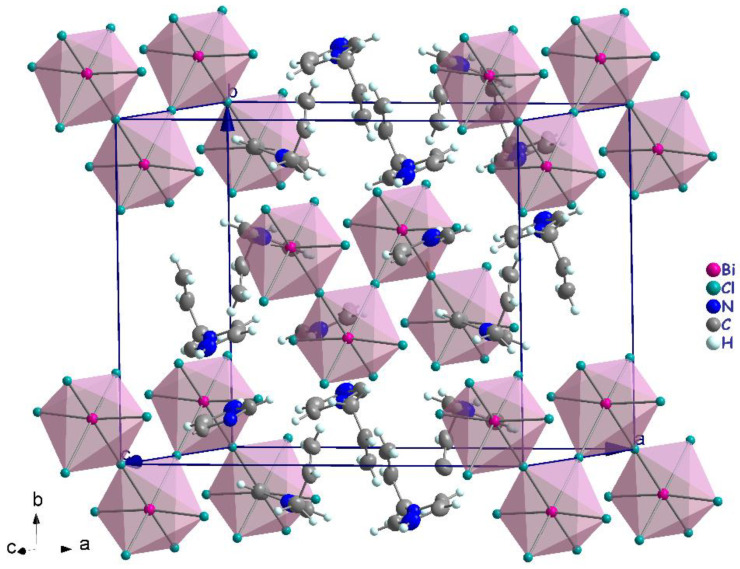
Crystal packing in the structure of (I).

The polymeric [BiCl_5_]^2−^ anion comprises distorted BiCl_6_ octahedra that form one-dimensional zigzag chains by sharing the cis corners Cl3 and Cl3^i^ (generated by the two-fold symmetry operation; i: −*x* + 1, *y*, −*z* + 1/2). The chains propagate along the [001] direction (Figure 4). The (C_6_H_9_N_2_)^+^ cations are inserted in the voids between the chains (Figure 4). The Bi–Cl distances involving the terminal chlorides (Bi–Cl1 = 2.559 (6) Å and Bi–Cl2 = 2.698 (7) Å, are predictably shorter than those involving the bridging ones (i.e., Bi1–Cl3 = 2.968 (2) Å). These values are in the range characteristic of pentachlorobismuthates and are close to the values found for (C_2_H_7_N_4_O)_2_[BiCl_5_] [39] (2.546 (3)–2.881 (3) Å) and for [NH_3_(CH_2_)_6_NH_3_]BiCl_5_ [40] (2.574 (2)−2.878 (2) Å). The Cl–Bi–Cl bond angles in (I) range from 86.85 (2) ° to 94.27 (4) ° for cis and 171.86 (3) ° to 180 ° for trans arrangements, which suggests a distortion of the [BiCl_6_]^3−^ octahedra (Table 2). In (C_2_H_7_N_4_O)_2_[BiCl_5_] [39], the Cl–Bi–Cl bond angle is 148.77 ° (10). This distortion is likely due to the fact that the amines are hydrogen-bonded to both bridging and apical Cl atoms from one chain. Moreover, in the title compound, imidazole forms strong N–H**⋯**Cl interactions only with apical Cl acceptors within one [BiCl5]_∞_^2−^ chain.

Examination of the geometrical features of the organic moiety shows that the 1-allylimidazolium cation exhibits a non-regular configuration. The plane of the allyl group makes a dihedral angle of 84.63° (16) with the plane of the imidazole ring and the twist of the allyl group (torsion N1–C3–C2=C1) is −2.1 ° (5)). This configuration is due to intra-molecular hydrogen-bonding interactions C1–H1A**⋯**N1. Such a result is not similar to the known data for allylimidazolium cations in homologous compounds [41,42]. Indeed, in Bi_4_I_16_.4(C_6_H_9_N_2_). 2(H_2_O) [42], the allyl group has regular configuration where the dihedral angle between the allyl group and imidazole ring is 77.9 ° and the torsion N1–C1–C4=C6 is 129.2 ° (2).

**Figure 5:**
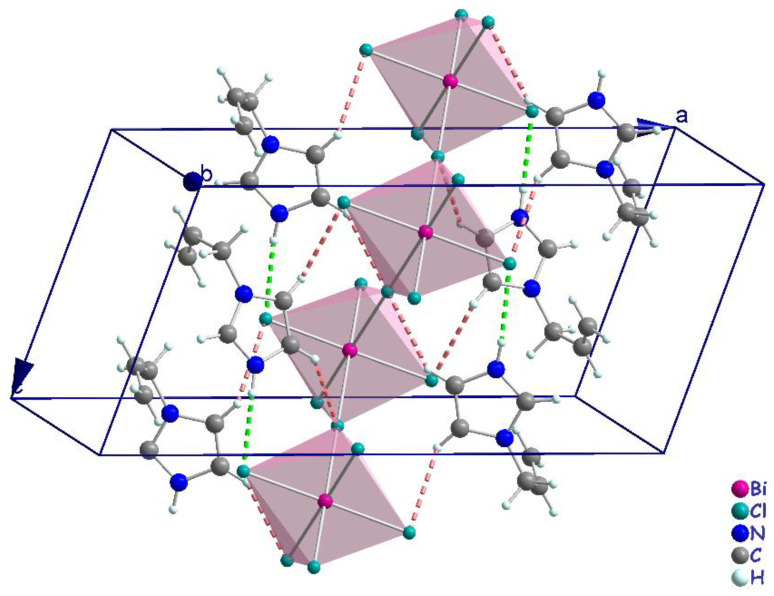
Projection representing the N–H···Cl (green dashed line) and C–H···Cl (pink dashed line) hydrogen-bonding interactions between anions and cations.

**Figure 6:**
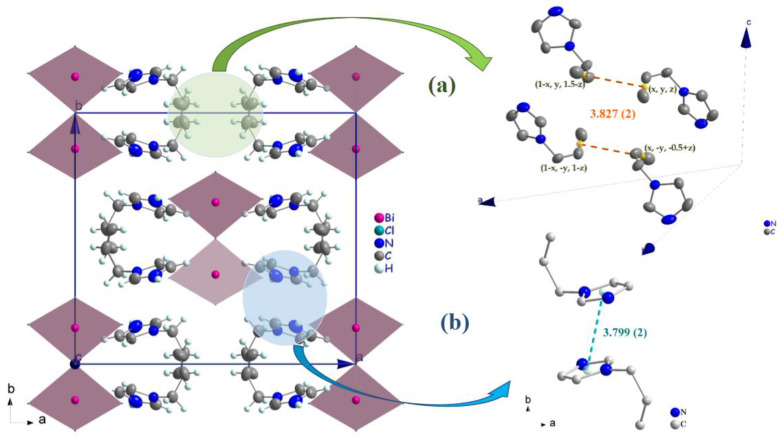
(**a**) π(C=C)**^...^**π(C=C) interactions (orange broken lines) between allyl groups in the cations (**b**) parallel π–π -stacking interactions (cyan broken lines) in the cation.

Generally, the crystal structure cohesion is achieved via various types of non-covalent interactions. The principal feature of interest in this structure is hydrogen bonding (Figure 5), which contributes to the stabilization of the crystal packing. As shown in Figure 5, the N–H and C–H moieties in the (C_6_H_9_N_2_)^+^ cation act as hydrogen-bond donors with chlorine vertices of [BiCl_5_]^2−^ anions (Table 3). The presence of π–π stacking between imidazolium rings and unusual π(C=C)**^...^**π(C=C) interactions shows another kind of interaction [43]. The measurement of their forces is determined by the centroid distance between the neighboring imidazolium ring (d = 3.779 (2) Å) and the interacting distance measured from the middle of the aromatic C–C bond (*x*, *y*, *z*; 1 − x, *y*, 1.5 − *z*; 1 − *x*, −*y*, 1 − *z*; *x*, −*y*,−0.5 + *z*) is d = 3.827 (2) Å, which demonstrates the presence of weak π–π stacking (Figure 6a,b).

### 3.3. Hirshfeld Surface Analysis, Two-Dimensional Fingerprint Plots and Enrichment Ratios (E_XY_)

**Figure 7:**
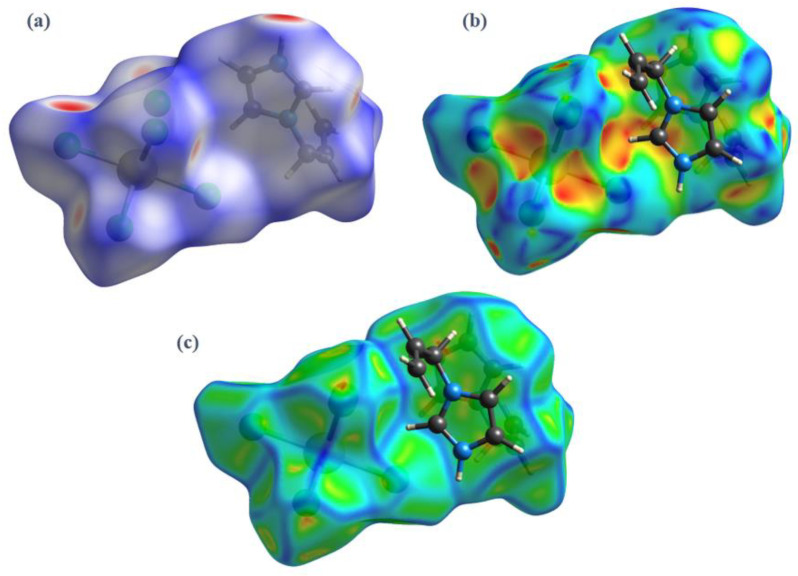
3D Hirshfeld surfaces of (I) mapped with (**a**) d_norm_, (**b**) shape index, and (**c**) curvedness.

The Hirshfeld surfaces of (I) were mapped over d_norm_, curvedness, and shape index (Figure 7a–c).

**Figure 8:**
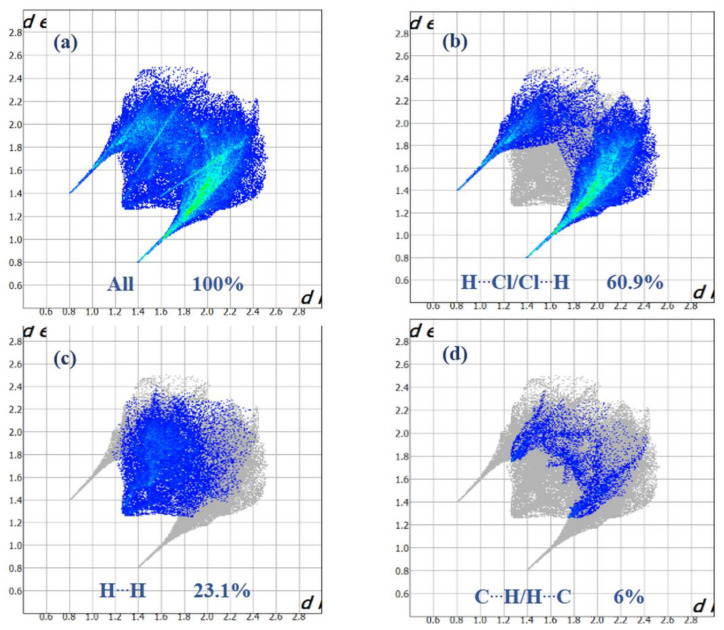
Selected fingerprint plots for (I) delineated into (**a**) All, (**b**) Cl**⋯**H, (**c**) H**⋯**H, (**d**) and C**⋯**H contacts.

Intense red regions in the surface mapped over d_norm_ indicate that close contact interactions are apparent around the chlorine, nitrogen and carbon atoms participating in N–H···Cl and C–H···Cl hydrogen bonds (Table 1) [34]. The curvedness and shape-index provide further chemical understanding into the molecular arrangement. A surface with low curvedness designates a flat region, and it is indicative of π–π stacking interactions in the crystal. The donor and the acceptors of π–π stacking can be known as blue and red triangles around the participating atoms on the surfaces mapped over shape-index properties corresponding to H···H contacts, as shown in Figure 7b. The overall fingerprint plots are calculated, including all intermolecular contacts, and the decomposed fingerprint plots, which focus on specific interactions (Figure 8). Relative contributions to the Hirshfeld surface for all intermolecular contacts in (I) are shown in Figure S2. Globally, the H**⋯**Cl contacts are the most favored interactions, and their relative contribution reaches 60.9% (Figure 8a). There are indeed three N2–H7···Cl2^iii^, C4–H4···Cl1^iv^ and C5–H5···Cl3^v^ hydrogen bonds in the crystal structure (Table 3). These contacts are the most frequent interactions due to the abundance of chlorine and hydrogen on the molecular surface (% S_Cl_ = 33.45% and % S_H_ = 57.9%) (Table 4). The fingerprint plot decomposition shows that H**⋯**H contacts comprise 23.1% of the total Hirshfeld surface area (Figure 8b) and are the second most frequent interactions due to the abundance of hydrogen on the molecular surface (57.9%). The C**⋯**H contacts represent the third most important interaction on the surface, with a percentage around 6% (Figure 8c) and an enrichment ratio higher than the unit E_C_**⋯**_H_ = 1.13 (Table 4). The N**⋯**C and C**⋯**C contacts are favored and display enrichment values (E_N_**⋯**_C_ = 6.87 and E_C_**⋯**_C_ = 2.85) (Table 4), and they represent a major attraction in the crystal despite the small surface areas of carbon and nitrogen (4.6% and 1.7% of the total surface). This type of contact corresponds to the π–π interactions between cationic molecules (the distance between the centroids of two antiparallel organic cations is 3.799 (2) Å and the distance between centroids of two parallel allyl groups in the cations is 3.287 (2) Å). The [BiCl_5_]^2−^ octahedra also contributes to the molecular surface and displays enrichment values (E_Bi_**⋯**_Cl_ = 2.43). The Cl**⋯**Cl contacts are the third most frequent interactions (11.19%) (Table 4) due to the abundance of chlorine on the molecular surface (33.45%). However, these contacts are highly diminished with an enrichment ratio around 0.07 (Table 4). Finally, the five types of contacts contribute significantly to the stability of the crystal structure. In conclusion, this analysis for intermolecular interactions is consistent with those observed by X-ray diffraction analysis.

### 3.4. Vibrational Properties

**Figure 9:**
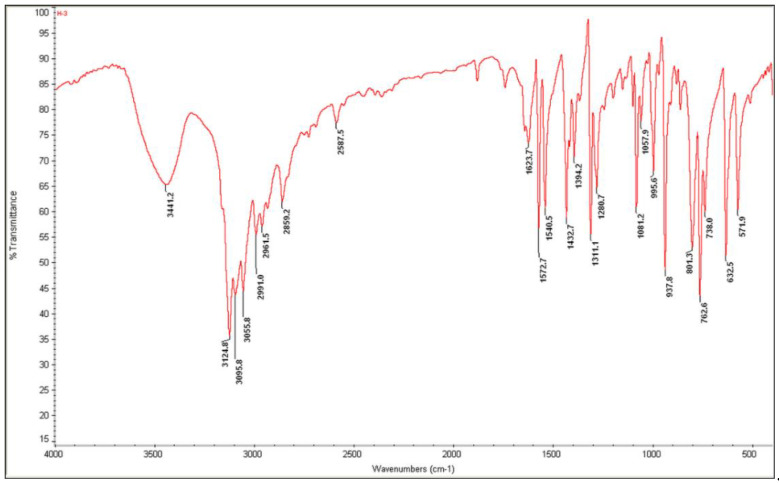
Experimental infrared spectrum of (I).

**Figure 10:**
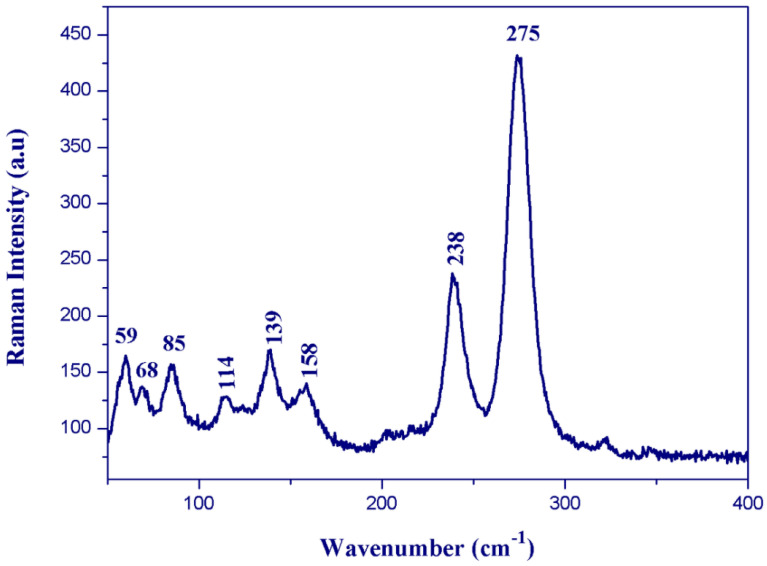
Single crystal Raman spectrum of (I).

All assignments are based on the spectra of the previous study of 1-allylimidazolium cation [41,44,45,46]. The experimental FTIR and FT-Raman vibrational spectra are shown in Figure 9 and Figure 10, respectively.

The Raman bands observed below 400 cm^−1^ for (I) (Figure 10) correspond to the anions’ internal vibrational modes. The bands observed between 300 and 125 cm^−1^ on the Raman spectrum were assigned to the Bi–Cl stretching modes. The intense band observed at 275 cm^−1^ was assigned to the terminal Bi–Cl bonds’ stretching mode, whereas the weak band located at 238 cm^−1^ was assigned to the stretching mode of the bridging Bi–Cl bonds. The lattice vibrations were found at wavenumbers lower than 100 cm^−1^.

### 3.5. Optical Absorption

**Figure 11:**
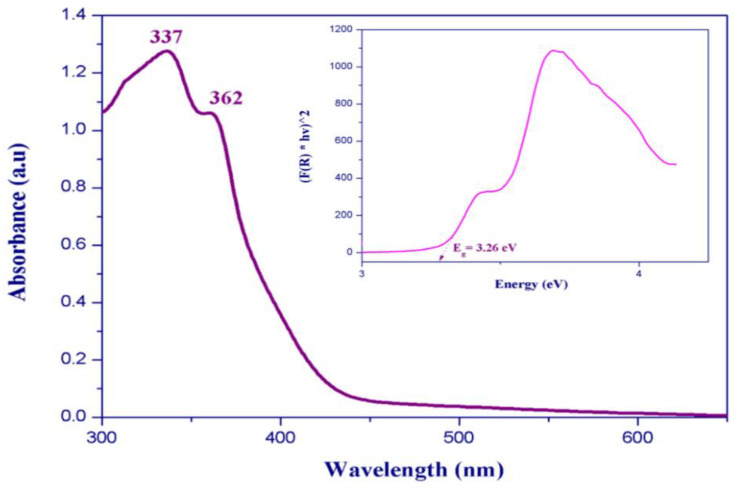
(**a**) Room temperature UV–Vis absorption spectrum and (**b**) diffuse reflectance spectrum in Kubelka–Munk units of (I).

Two absorption bands centered at 337 and 362 nm are observed in the room-temperature UV–Visible absorption spectrum (Figure 11). These bands are typically assigned to metal-centered (MC) transitions [47,48,49] and to the ligand-to-metal charge transfer transitions [50,51]. We measured the UV–Visible diffuse reflectance spectra at room temperature to determine the optical band gap of (C_6_H_9_N_2_)_2_BiCl_5_. The absorbance as a function of reflectance is given by the Kubelka–Munk theory (Equation (1)):

(1) $$F\left(R\right)\;=\;\alpha \;=\;\frac{\left(1-R\right)}{\left(2R\right)}$$

The energy band gap obtained by extrapolation of the linear portion of the absorption edges was estimated to be 3.26 eV, indicating a semiconductor nature of the title compound.

### 3.6. Density Functional Theory Calculations

To understand the title compound’s optical properties, we have determined the electronic structure, the density of state, and optical properties such as the dielectric function and the refractive index. Stability of the title compound depends primarily on the organic part; therefore, we calculated the HOMO/LUMO energy gap and the Fukui indices of the imidazolium cation.

#### 3.6.1. Band Structure and Density of States

**Figure 12:**
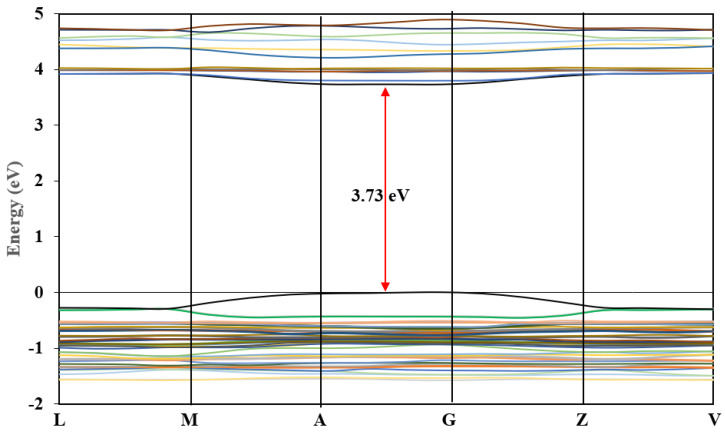
Band structure of (I).

The calculated band structure of (C_6_H_9_N_2_)_2_BiCl_5_ is shown in Figure 12. The maximum of the highest occupied valence bands and the minimum of the lowest unoccupied conduction bands lay at the same point (between A- and G-points) (Figure 12); therefore, the title compound is a direct semiconductor. The band-gap value was about 3.73 eV (Figure 12). This calculated value is higher than the experimental optic gap determined from the UV–Vis absorption spectrum (Figure 11). This difference may be due to the self-interaction error of the exchange and correlation (XC) functional [52,53,54].

**Figure 13:**
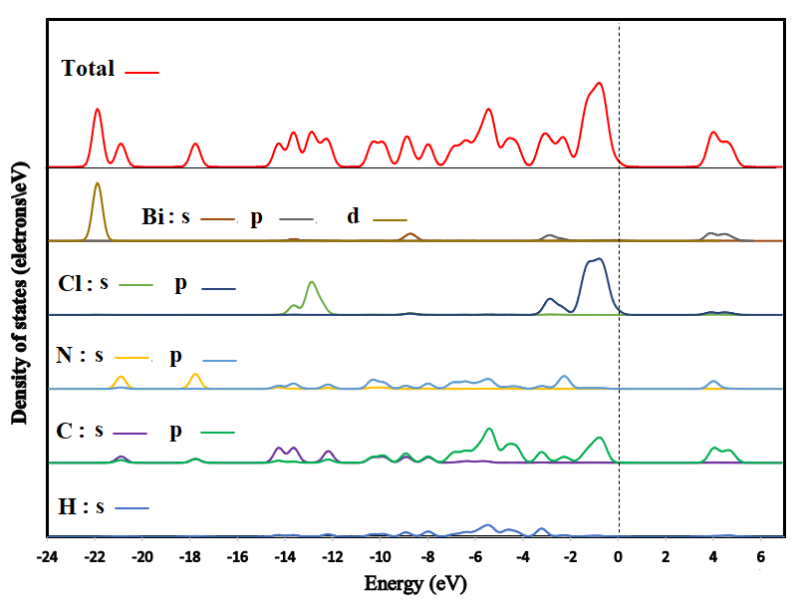
Density of states of (I).

We illustrated the total and partial densities of states of (C_6_H_9_N_2_)_2_BiCl_5_ in Figure 13. The Fermi level (showed by a dotted line) is set to zero (EF). Here, we have treated H: 1s^1^; C: 2s^2^2p^2^; N: 2s^2^2p^3^; Cl: 3s^2^3p^5^ and Bi: 6s^2^4f^14^5d^10^6p^3^ as valence electrons. Low-lying bands located within the energy range between −23 and −20 eV below the Fermi level mainly consist of Bi-5d and C-2s, and N-2s orbitals. The highest occupied crystal orbital is a mixture of Cl-3p and C-3p. With the majority from Cl-3p, conversely, the lowest unoccupied crystal orbital located above 3.73 eV is a mixture of Bi-6p and C-2p states with a smaller contribution of N-2p and Cl-3p states.

#### 3.6.2. Optical Properties

The title compound’s optical properties can be described by the complex dielectric function ε(ω), which represents the linear response of the system to an external electromagnetic field with a small wave vector. The optical properties are also associated with electron mobility and recombination rate of the electron [55]. We can express it as:

ε (ω) = ε1(ω) + iε2(ω)(2)

The imaginary part of the complex dielectric function can be expressed as:

(3) $$\varepsilon_{2}\left(\omega \right)=\left(\frac{4\pi^{2}e^{2}}{m^{2}\omega^{2}}\right)\underset{Ij}{\sum }\int \langle i|M|J\rangle l^{²}f_{i}(1-f_{i})\delta \left(E_{f}-E_{i}-\omega \right)d^{3}k\;\;\;\;\;\;$$

where *M* is the dipole matrix element; *i* and *j* are the initial and final states, respectively; and *f_i_* is the Fermi distribution function for the *i*th state. *E_i_* is the energy of an electron in the *i*th states. By utilizing the Kramers Kronig relationship [56,57,58], the real part, ε1(ω), can be determined from the imaginary part as:

(4) $$\varepsilon_{1}\left(\omega \right)=1+\frac{2}{\pi }p\;\overset{\infty }{\underset{0}{\int }}\frac{\omega^{\prime }\varepsilon_{2}\left(\omega^{\prime }\right)d\omega^{\prime }}{\omega^{{}^{\prime }2}-\omega^{2}}$$

where *p* indicates the principal value of the integral.

**Figure 14:**
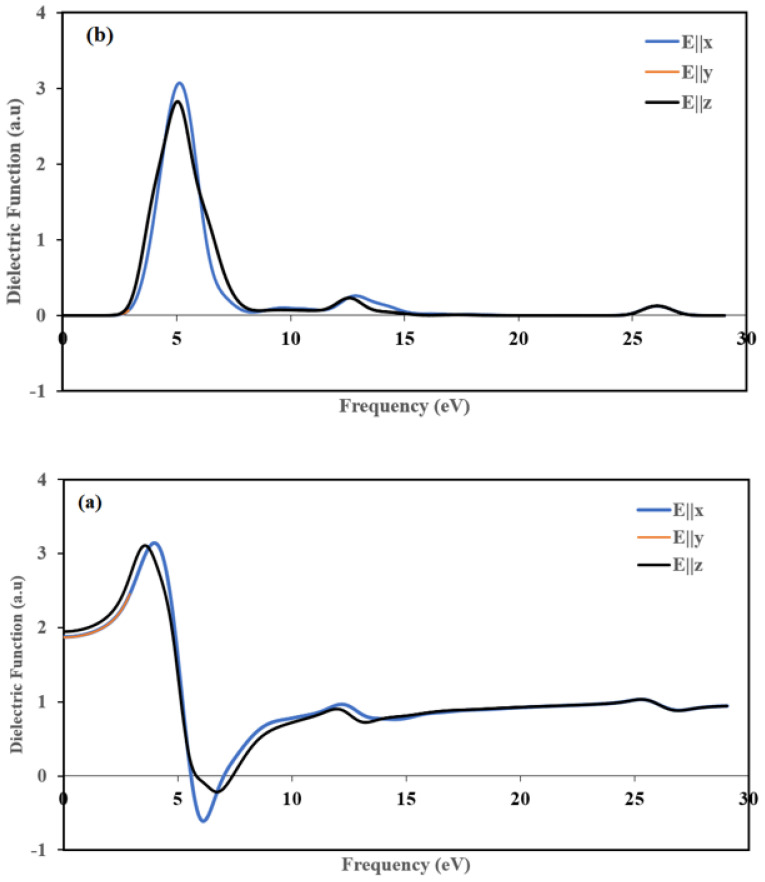
Calculated (**a**) real ε1 and (**b**) imaginary ε2 parts of the dielectric function, in the three directions [100], [010] and [001].

The real part ε1(ω) of the complex function as a function of the used energy from 0 to 29 eV in three directions is shown in Figure 14a. The static functions ε1(0) of the real part is 1.87 for E||*x* and E||*y* and 1.94 for E||*z*. The two plots of the ε1(ω) along (100) and (010] directions are stackable, and they are different from the (001 direction, which means that the title compound is anisotropic. The maximum ε1(ω) peak is progressively increased by increasing the photon energy at around 4, 4, and 3.5 eV for E||*x*, E||*y*, and E||*z*, respectively. These peaks occur due to the gradual transition of mobile electrons from the topmost of the valance to the conduction band’s visible bottom [59]. The real part ε1(ω) becomes zero at about 5.58 eV along the [100] and [010] directions and 5.83 eV along the [001] direction, whereas at high frequencies, the zero crossings of ε1(ω) corresponds to the location of the screened plasma frequency which is situated at 7.02 eV, 7.02 eV and 7.42 eV for polarization directions [001], [010] and [001], respectively. At 25.5 eV, ε1(ω) occurs at the same peak in the three polarization directions. 

We illustrated imaginary part plots of the dielectric function ε2(ω) in Figure 14b. The variations are the same along [100] and [010] directions, and they are different from the [001] direction, which indicates that the title compound displays anisotropy. The maximum values were around 5.15, 5.15, and 5.04 eV for E||*x*, E||*y* and E||*z*, respectively. These peaks depicted in Figure 14(b) belong to an energy transition between some orbitals corresponding to certain energy because ε2(ω) is related to the density of state.

The optical constants such as refractive index *η*(*ω*) and the extinction coefficient *k*(*ω*), are calculated in terms of the real and the imaginary parts of the complex dielectric function as follows [60]:

(5) $$\;\eta \left(\omega \right)=\frac{\sqrt{\varepsilon_{1}^{2}\left(\omega \right)+\varepsilon_{2}^{2}\left(\omega \right)}+\varepsilon_{1}^{\frac{1}{2}}\left(\omega \right)}{\sqrt{2}}$$

(6) $$\;k\left(\omega \right)=\frac{\sqrt{\varepsilon_{1}^{2}\left(\omega \right)+\varepsilon_{2}^{2}\left(\omega \right)}-\varepsilon_{1}^{1/2}\left(\omega \right)}{\sqrt{2}}$$

**Figure 15:**
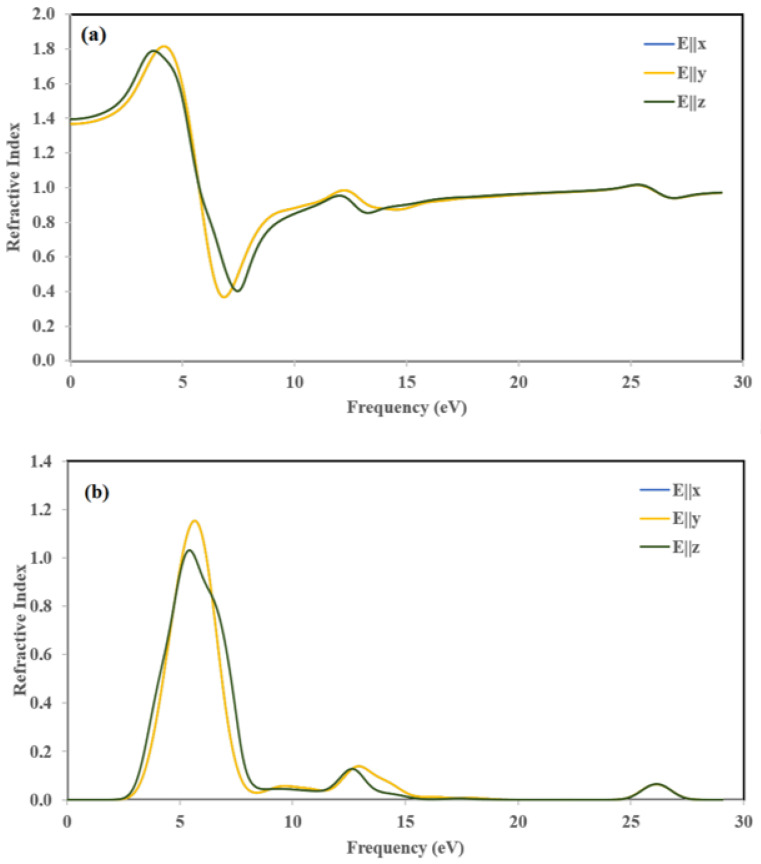
Calculated (**a**) refractive index *η*(*ω*) and (**b**) extinction coefficient *k*(*ω*).

The variations of the refractive index *η*(*ω*) and extinction coefficient *k*(*ω*) are calculated according to the three crystallographic directions. The results are displayed in Figure 15. The variation of *η*(*ω*) and *k*(*ω*) along the [100] and [010] directions are the same. Thus, for the following part, we discuss only [100] and [001] polarization directions.

The refractive index *η*(*ω*) (Figure 15a) represents the ratio of denser to the rare medium. The static refractive index values with considerable potentials are 1.37, and 1.39 for E||*x* and E||*z*, respectively. The maximum peaks of refractive index *η*(*ω*) were obtained at 4.2 and 3.7 eV for E||*x*, and E||*z*, respectively. The magnitude of refractive spectra increased due to less band-gap and by increasing photon energy.

The extinction coefficients (*k*(*ω*)) (Figure 15b) increased drastically to 1.15 and 1.03 at about 5.64 eV and 5.34 eV along the [100] and [001] directions and then decreased rapidly to the minimum value at about 8.35 eV, which is the indication of small absorption in this spectral region. It is worth pointing out that the extinction coefficient *k*(*ω*) is larger than the refractive index *η*(*ω*) in the spectral region from 5.64 eV until 8.35 eV, which means that light cannot propagate in this region.

#### 3.6.3. Frontier Molecular Orbital

The Frontier molecular orbitals provide the nature of reactivity and some of the molecules’ physical and structural properties. Both HOMO and LUMO are the main orbitals considered in the study of the chemical reactivity of the molecule. The HOMO energy characterizes the electron-donating ability; however, the electron-accepting ability is characterized by LUMO energy. Obviously, the energy difference between HOMO and LUMO orbitals, called the energy gap, characterizes the molecule’s chemical stability [61]. The energy gap is largely responsible for the chemical and spectroscopic properties of the molecules [62].

**Figure 16:**
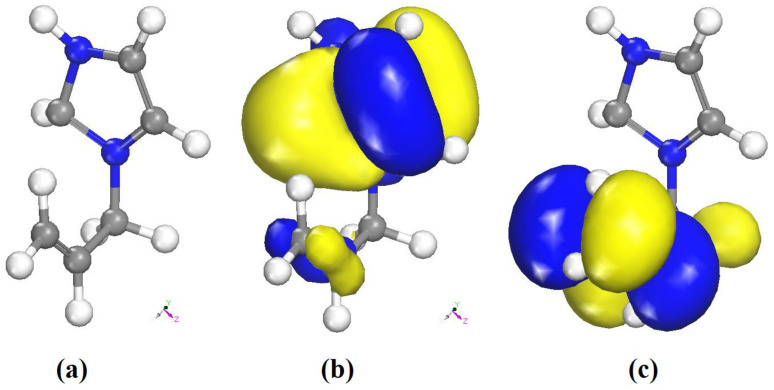
(**a**) Optimized molecular structure, (**b**) frontiers orbitals distribution HOMO, and (**c**) LUMO of the organic part of (I).

The optimized geometry of the organic part of the title compound obtained from the DFT calculations is shown in Figure 16a. The HOMO and LUMO orbitals are shown in Figure 16b,c. The positive and negative phases are represented in blue and yellow color, respectively.

E_HOMO_ = −0.7587 eV

E_LUMO_ = −0.9689 eV

ΔE_gap_ = 0.2102 eV

The low HOMO/LUMO energy gap of the organic cation is consistent with a high reactivity. At this level of calculation, the HOMO is mainly localized on the imidazolium ring, while the LUMO has a contribution from the allyl moiety only. The ionization potential (IP), electron affinity (EA), Mulliken electronegativity (*χ*) and absolute hardness (*η*) can be deduced from the values of E_HOMO_ and E_LUMO_ as follows. The ionization potential (IP) and electron affinity (EA) are related directly to E_HOMO_ and E_LUMO_ using Equations (7) and (8) [63]:

IP = −E_HOMO_ = 0.7587 eV (7)

EA = −E_LUMO_ = 0.9689 eV (8)

However, the Mulliken electronegativity (*χ*) and absolute hardness (*η*) can be approximated using Equations (9) and (10) [64,65]:

(9) $$\chi =\frac{IP+EA}{2},\chi =-\frac{E_{LUMO}+E_{HOMO}}{2}=0.8638\;\mathrm{eV}$$

(10) $$\eta =\frac{IP-EA}{2},\eta =-\frac{E_{LUMO}-E_{HOMO}}{2}=0.1051\;\mathrm{eV}$$

#### 3.6.4. Fukui Function

The Fukui function (FI) is an important tool to determine the regioselectivity of the organic molecule and its reactive regions in terms of nucleophilic (f^+^) and electrophilic attack (f^−^) [66]. The Fukui indices as function of the atomic charges are given by:

(11) $$f_{k}^{-}=\;\left(N\right)\;-\;\left(N-1\right)\;\left(\mathrm{for}\;\mathrm{electrophilic}\;\mathrm{attack}\right)$$

(12) $$f_{k}^{+}=\;\left(N\;+\;1\right)\;-\;\left(N\right)\;\left(\mathrm{for}\;\mathrm{nucleophilic}\;\mathrm{attack}\right)$$

(13) $$f_{k}^{0}\;=\;[q_{k}\;(N\;+\;1)\;-\;q_{k}\;(N-1)]/2\;(\mathrm{for}\;\mathrm{radical}\;\mathrm{attack})$$

where q_k_ is the electronic charge of atom k and N is the number of electrons. The condensed Fukui function values ($f_{k}^{-}$, $f_{k}^{+}$, $f_{k}^{0}$) were calculated for electrophilic, nucleophilic, and radical attacks have been performed using Dmol^3^ code.

Table 5 shows that C(5), identified in Figure 7 by the red circle, has the highest values ${\;f}_{k}^{+},\;f_{k}^{-}$ and $f_{k}^{0}$. Thus, this carbon atom is the most favorite site for electrophilic, nucleophilic, and radical attack. The results obtained from the Fukui function and the analysis of the LUMO and HOMO orbitals (Figure 7b,c) are in good agreement, and the two methods lead to the same predictions of most electron-deficient site.

## 4. Conclusions

The (C_6_H_9_N_2_)_2_BiCl_5_ hybrid is obtained via slow evaporation at room temperature. This organic–inorganic material was crystallized in the monoclinic space group C2/c. Hydrogen bonds connected the organic and inorganic components. Scanning electronic microscopy (SEM) and energy-dispersive X-ray (EDX) were carried out. The Hirshfeld contact analysis implies that the enriched Cl**⋯**H, H**⋯**H, and C**⋯**H hydrogen bonds are the driving forces in the molecular arrangement and in the formation of the crystal packing. The optical absorption revealed that the band-gap of this compound is 3.26 eV. The characteristic vibrational peaks clearly appeared in FTIR and Raman spectra. The DFT calculation supports the experimental data and shows that the title compound cannot be used for photovoltaic cell because of its high optical gap (3.26 eV) and the low stability of the organic part (ΔE_gap_ = 0.2102 eV).

## Figures and Tables

**Table 1:** Crystallographic data and structure refinement parameters for C_6_H_9_N_2_)_2_BiCl_5_ (I).

Empirical Formula	BiCl_5_·2(C_6_H_9_N_2_)
Formula weight (g/mol)	604.53
Crystal system, space group	Monoclinic, C2/c
a (Å)	17.0571 (10)
b (Å)	14.3209 (9)
c (Å)	8.5420 (6)
β (°)	109.760 (2)
V (Å^3^)	1963.7 (2)
Z	4
µ (mm^−1^)	9.66
Dx (Mg∙m^−3^)	2.045
F(000)	1144
Crystal size (mm)	0.42 × 0.11 × 0.08
Crystal habit	column, colorless
θmin/θmax (°)	2.538/28.344
Measured reflections	44657
Independent reflections	2454
Observed reflections with I > 2σ(I)	2362
R_int_	0.053
Data/restraints/parameters	2454/0/103
R[F2 > 2σ(F^2^)]	0.015
wR(F^2^)	0.037
GooF = S	1.15
Δρmax/Δρmin (e Å^−3^)	1.60/−0.68

**Table 2:** Selected bond distances and angles of (I).

**Bond Distances (Å)**	
Bi1–Cl1	2.559 (6)
Bi1–Cl2	2.697 (7)
Bi1–Cl3	2.968 (1)
**Bond Angles (°)**	
Cl1^i^–Bi1–Cl1	94.27 (4)
Cl1–Bi1–Cl2^i^	87.61 (2)
Cl1–Bi1–Cl2	86.85 (2)
Cl2^i^–Bi1–Cl2	171.86 (3)
Cl1^i^–Bi1–Cl3	86.85 (2)
Cl2–Bi1–Cl3	93.13 (2)
Cl1^i^–Bi1–Cl3^i^	178.88 (2)
Cl1–Bi1–Cl3^i^	93.13 (2)
Cl2–Bi1–Cl3^i^	86.85 (2)
Cl3–Bi1–Cl3^i^	92.025(6)
Bi1^ii^–Cl3–Bi1	180

Symmetry codes: (i) −*x* + 1, *y*, −z + 1/2; (ii) −*x* + 1, −*y* + 1, −*z*.

**Table 3:** Hydrogen-bond geometry (Å, °) for (I).

*D*–H···*A*	*D*–H	H···*A*	*D*···*A*	*D*–H···*A*
N2–H7···Cl2 ^iii^	0.86	2.35	3.190 (3)	165
C4–H4···Cl1 ^iv^	0.93	2.75	3.611 (4)	155
C5–H5···Cl3 ^v^	0.93	2.77	3.557 (3)	143
C1–H1A···N1	0.93	2.53	2.853(5)	101
C6–H6···Cl2	0.93	2.84	3.528 (3)	132

Symmetry codes: (iii) *x*, *y*, *z* − 1; (iv) *x* + 1/2, −*y* + 3/2, *z* − 1/2; (v) −*x* + 1, *y*, −*z* − 1/2.

**Table 4:** Hirshfeld contact surfaces (grey), derived “random contact” (blue) and “enrichment ratios” (green) for compound (I).

Atoms	H	C	N	Cl	Bi
**Surface (%)**	57.9	4.6	1.7	33.45	2.15
**H**	**33.52**	-	-	**% contacts**	-
**C**	5.33	0.21	-	-	-
**N**	1.97	0.16	0.03	-	-
**Cl**	**38.85**	3.09	1.14	**11.19**	-
**Bi**	**2.49**	0.20	0.07	1.44	0.05
**H**	0.69	-	-	**Enrichment**	-
**C**	**1.13**	**2.85**	-	-	-
**N**	0.96	**6.87**	6.66	0.0	-
**Cl**	**1.57**	0.29	0.0	0.07	-
**Bi**	0.32	0.0	0.0	**2.43**	0.0

**Table 5:** Calculated Fukui indices of the organic part of (I).

	$f_{k}^{+}$	$f_{k}^{-}$	$f_{k}^{0}$
**N1**	0.045	0.044	0.045
**C6**	0.071	0.093	0.082
**H6**	0.064	0.061	0.063
**C1**	−0.011	0.017	0.003
**H1A**	0.022	0.024	0.023
**H1B**	0.025	0.018	0.021
**C2**	0.011	−0.003	0.004
**H2**	0.036	0.031	0.033
**C3**	−0.011	−0.012	−0.011
**H3A**	0.039	0.040	0.040
**H3B**	0.049	0.038	0.043
**C5**	0.247	0.237	0.242
**H5**	0.147	0.134	0.141
**N2**	0.069	0.104	0.087
**H7**	0.074	0.063	0.068
**C4**	0.057	0.053	0.055
**H4**	0.065	0.057	0.061

## Supplementary Materials

The following are available online at https://www.mdpi.com/1422-0067/22/4/2030/s1. Figure S1: Micrograph SEM image of (I); Figure S2: Relative contributions to the Hirshfeld surface for all intermolecular contacts in (I); Table S1: Unit cell parameters of the single crystal and the optimized structure; Table S2: The atomic coordinates in the structure S1 and S2; Table S3: The atomic displacements from the starting set to the optimized structure; Table S4: Evaluation of the structure similarity.
