# Supplementary material for: One-Dimensional Organic–Inorganic Material (C6H9N2)2BiCl5: From Synthesis to Structural, Spectroscopic, and Electronic Characterizations
Source: Int J Mol Sci. 2021 Feb 18;22(4):2030. doi: 10.3390/ijms22042030 (PMC7922144; doi:10.3390/ijms22042030)
Supplement: Supplementary file 1 [file ijms-22-02030-s001.pdf]

Supplementary material of One-Dimensional organic-inorganic material $(C_6H_9N_2)_2BiCl_5$: From synthesis to structural, spectroscopic and electronic characterizations

Hela Ferjani [1,*], Hammouda Chebbi [2] and Mohammed Fettouhi [3]

Corresponding Author: hhferjani@imamu.edu.sa

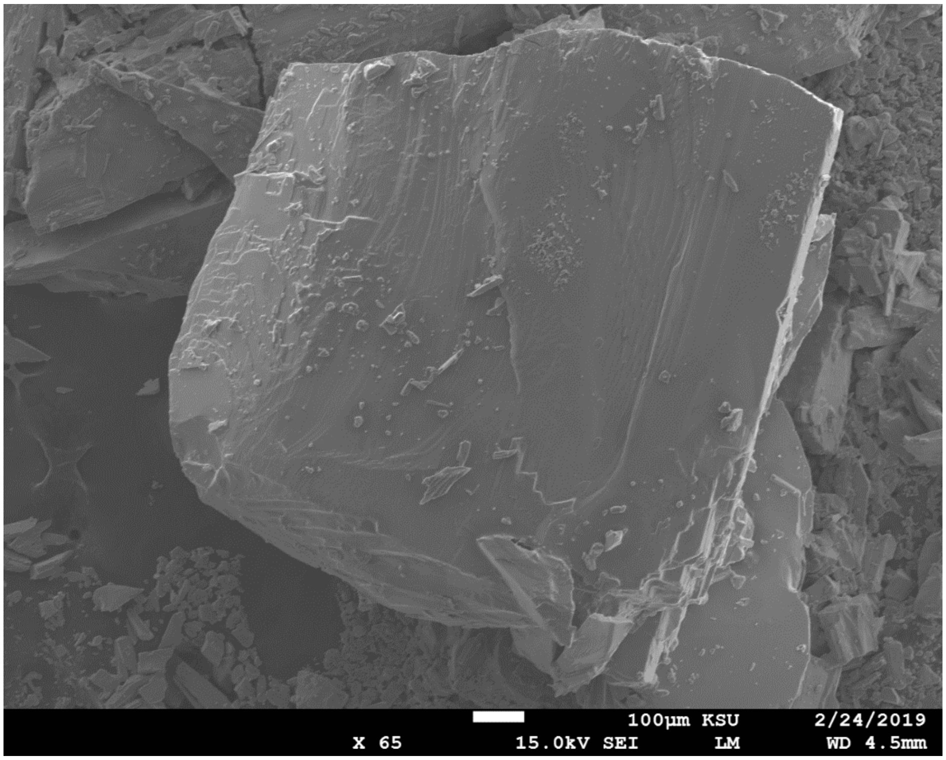

Figure S1. Micrograph SEM image of (I)

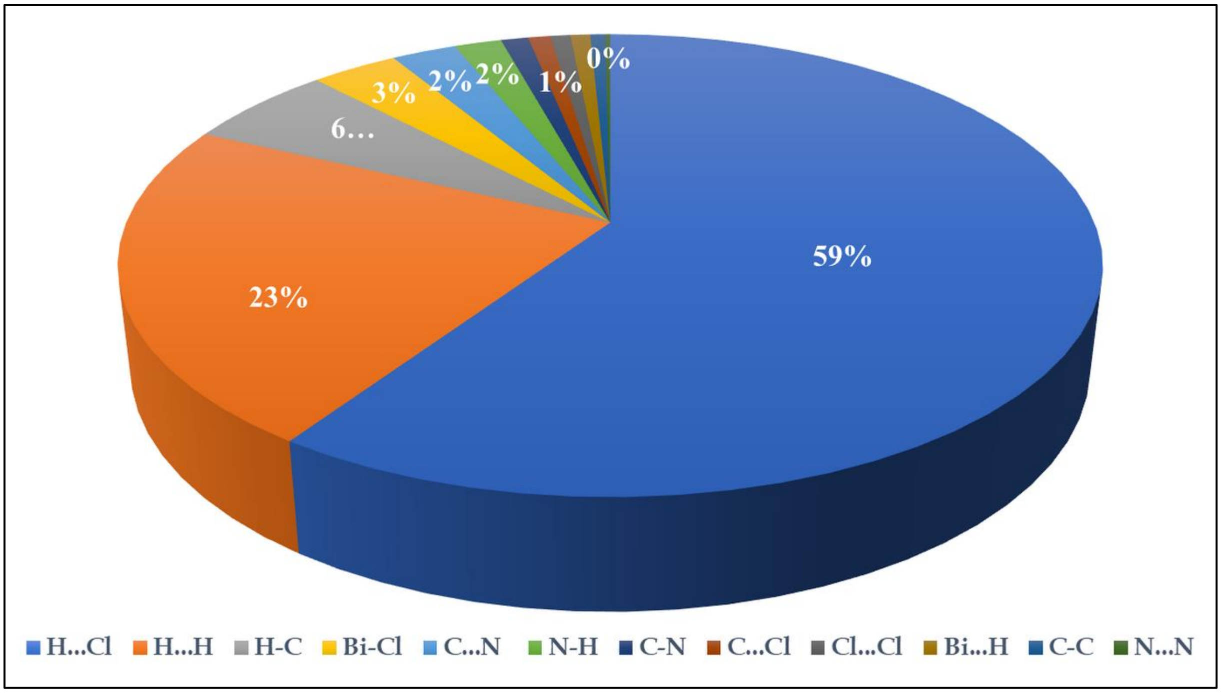

Figure S2: Relative contributions to the Hirshfeld surface for all intermolecular contacts in (I).

Table S1: Unit cell parameters of the single crystal and the optimized structure

S1	single crystal	17.0571	14.3209	8.5420	90.	109.7600	90
S2:	optimized structure	17.4069	15.3354	8.7387	90.	109.2583	90

Table S2: The atomic coordinates in the structure S1 et S2

**Atom Mappings**					
**WP**		**Atom**	**Coordinates in S_1**	**Atom**	**Coordinates in S_2**
8f	(x,y,z)	Cl1	(0.500190,0.765490,0.469680)	Cl1	(0.498850,0.750660,0.459120)
8f	(x,y,z)	Cl2	(0.667610,0.657300,0.365500)	Cl2	(0.664660,0.640810,0.360120)
8f	(x,y,z)	N1	(0.779340,0.632450,0.020100)	N1	(0.778420,0.633960,0.002950)
8f	(x,y,z)	C5	(0.659300,0.612900,0.826700)	C5	(0.659100,0.609560,0.813280)
8f	(x,y,z)	H5	(0.603800,0.598200,0.769500)	H5	(0.597260,0.592470,0.740860)
8f	(x,y,z)	C1	(0.871600,0.464200,0.126100)	C1	(0.876240,0.479290,0.124290)
8f	(x,y,z)	H11	(0.827500,0.465100,0.025900)	H11	(0.826910,0.472550,0.008960)
8f	(x,y,z)	H12	(0.902600,0.409900,0.160600)	H12	(0.914240,0.421930,0.168250)
8f	(x,y,z)	C2	(0.890010,0.538400,0.217700)	C2	(0.890180,0.553490,0.208790)
8f	(x,y,z)	H2	(0.934700,0.533900,0.316800)	H2	(0.939750,0.557590,0.324560)

8f	(x,y,z)	C3	(0.846700,0.630700,0.180500)	C3	(0.844020,0.637670,0.161100)
8f	(x,y,z)	H31	(0.824200,0.645600,0.267600)	H31	(0.816030,0.656810,0.252120)
8f	(x,y,z)	H32	(0.887100,0.678600,0.181100)	H32	(0.885790,0.690550,0.155520)
8f	(x,y,z)	C4	(0.787100,0.660000,0.878200)	C4	(0.786920,0.653750,0.858590)
8f	(x,y,z)	H4	(0.835300,0.683300,0.864800)	H4	(0.841890,0.679090,0.841710)
8f	(x,y,z)	N2	(0.715000,0.648960,0.759900)	N2	(0.714840,0.639320,0.742730)
8f	(x,y,z)	H7	(0.704500,0.662300,0.656600)	H7	(0.702250,0.646740,0.617750)
8f	(x,y,z)	C6	(0.699090,0.602600,0.988400)	C6	(0.698840,0.605980,0.976630)
8f	(x,y,z)	H6	(0.676700,0.579500,0.066000)	H6	(0.677760,0.587720,0.075940)
4e	(0,y,1/4)	Bi1	(0.500000,0.643940,0.250000)	Bi1	(0.500000,0.634970,0.250000)
4a	(0,0,0)	Cl3	(0.500000,0.500000,0.000000)	Cl3	(0.500000,0.500000,0.000000)

Table S3: The atomic displacements from the starting set to the optimized structure

WP		Atom	Atomic Displacements			
			u_x	u_y	u_z	|u|
8f	(x,y,z)	Cl1	-0.0013	-0.0148	-0.0106	0.2288
8f	(x,y,z)	Cl2	-0.0029	-0.0165	-0.0054	0.2426
8f	(x,y,z)	N1	-0.0009	0.0015	-0.0171	0.1436
8f	(x,y,z)	C5	-0.0002	-0.0033	-0.0134	0.1232
8f	(x,y,z)	H5	-0.0065	-0.0057	-0.0286	0.2461
8f	(x,y,z)	C1	0.0046	0.0151	-0.0018	0.2324
8f	(x,y,z)	H11	-0.0006	0.0075	-0.0169	0.1773
8f	(x,y,z)	H12	0.0116	0.0120	0.0076	0.2542
8f	(x,y,z)	C2	0.0002	0.0151	-0.0089	0.2295
8f	(x,y,z)	H2	0.0050	0.0237	0.0078	0.3508
8f	(x,y,z)	C3	-0.0027	0.0070	-0.0194	0.1855
8f	(x,y,z)	H31	-0.0082	0.0112	-0.0155	0.2241
8f	(x,y,z)	H32	-0.0013	0.0120	-0.0256	0.2725
8f	(x,y,z)	C4	-0.0002	-0.0063	-0.0196	0.1890

8f	(x,y,z)	H4	0.0066	-0.0042	-0.0231	0.2649
8f	(x,y,z)	N2	-0.0002	-0.0096	-0.0172	0.2008
8f	(x,y,z)	H7	-0.0022	-0.0156	-0.0389	0.3907
8f	(x,y,z)	C6	-0.0003	0.0034	-0.0118	0.1104
8f	(x,y,z)	H6	0.0011	0.0082	0.0099	0.1427
4e	(0,y,1/4)	Bi1	0.0000	-0.0090	0.0000	0.1285
4a	(0,0,0)	Cl3	0.0000	0.0000	0.0000	0.0000

NOTE: u_x, u_y and u_z are given in relative units. |u| is the absolute distance given in Å

Table S4: Evaluation of the structure similarity

**S**	**$d_{max.}$ (Å)**	**$d_{av.}$ (Å)**	**Δ**
0.0239	0.3907	0.2137	0.082

- **Lattice and atomic position criteria:**

- The degree of lattice distortion (**S**) is the spontaneous strain (sum of the squared eigenvalues of the strain tensor divided by 3). For the given two structures, the **degree of lattice distortion (S)** is **0.0239**.

- The maximum distance (**$d_{max.}$**) shows the maximal displacement between the atomic positions of the paired atoms. The **maximum distance ($d_{max.}$)** in this case is: **0.3907 Å**

- The arithmetic mean (**d_{av}**) of the distance. In this case, the **arithmetic mean (d_{av})** is **0.2137 Å**
.
- The measure of similarity (**Δ**) (Bergerhoff *et al.*, 1998) is a function of the differences in atomic positions (weighted by the multiplicities of the sites) and the ratios of the corresponding lattice parameters of the structures. The **measure of similarity (Δ)** calculated for this case is **0.082**.
